# Supplementary material for: Complexome profiling of the Chlamydomonas psb28 mutant reveals TEF5 as an early PSII assembly factor
Source: Plant Cell. 2025 Mar 17;37(6):koaf055. doi: 10.1093/plcell/koaf055 (PMC12164590; doi:10.1093/plcell/koaf055)
Supplement: koaf055_Supplementary_Data [file koaf055_supplementary_data.zip › psb28_interactive.zip!/plots/intensity/qualityPlots/peptides/_Cre17.g705900.t1.2.html]

### Cre17.g705900.t1.2

Trivial name:   
SeqCoverage [median]: 28.90  
Mass: 8.59 kDa  
  
 

| Sequence | MissedCleavage | Mass [Da] | wtIntensity | muIntensity | Score |
| --- | --- | --- | --- | --- | --- |
| AYTVAYER | 0 | 971.47 | 1008.72 | 852.14 | 95.53 |
| MVGIGEDMSELIER | 0 | 1577.74 | 1084.25 | 2794.06 | 110.66 |
|  |  |  | 2093.0 | 3646.2 |  |
